# Sustainable high-yield farming is essential for bending the curve of biodiversity loss

**DOI:** 10.1098/rstb.2023.0216

**Published:** 2025-01-09

**Authors:** Andrew Balmford, Ian J. Bateman, Alison Eyres, Tom Swinfield, Thomas S. Ball

**Affiliations:** ^1^ Department of Zoology, University of Cambridge, Cambridge, UK; ^2^ Conservation Research Institute, University of Cambridge, Cambridge, UK; ^3^ University of Exeter Business School, University of Exeter, Exeter, UK; ^4^ Land, Environment, Economics and Policy Institute, University of Exeter, UK

**Keywords:** biodiversity, land use, land sparing, farming, yield, food production

## Abstract

Food production does more damage to wild species than any other sector of human activity, yet how best to limit its growing impact is greatly contested. Reviewing progress to date in interventions that encourage less damaging diets or cut food loss and waste, we conclude that both are essential but far from sufficient. In terms of production, field studies from five continents quantifying the population-level impacts of land sharing, land sparing, intermediate and mixed approaches for almost 2000 individually assessed species show that implementing high-yield farming to spare natural habitats consistently outperforms land sharing, particularly for species of highest conservation concern. Sparing also offers considerable potential for mitigating climate change. Delivering land sparing nevertheless raises several important challenges—in particular, identifying and promoting higher yielding farm systems that are less environmentally harmful than current industrial agriculture, and devising mechanisms to limit rebound effects and instead tie yield gains to habitat conservation. Progress will depend on conservationists forging novel collaborations with the agriculture sector. While this may be challenging, we suggest that without it there is no realistic prospect of slowing biodiversity loss.

This article is part of the discussion meeting issue ‘Bending the curve towards nature recovery: building on Georgina Mace's legacy for a biodiverse future’.

## 1. Introduction

The scale and speed of the extinction crisis demand innovative thinking and bold responses. More than 50 years after recognizing the problem, and despite dozens of international agreements, near-daily calls to action and billions of dollars invested in conservation interventions, we are still collectively failing to bend the curve of biodiversity’s decline [1,2]. Continued business-as-usual conservation will not turn things around, and we are fast running out of time to change how we do things. Humanity has altered over 70% of the Earth’s land surface [3], halved the biomass stored in terrestrial vegetation [4], and impacted our fellow species so heavily that over one-quarter of those assessed are now threatened with extinction [5]. New work on the areas of habitat that individual species can occupy indicates that people have already reduced these by an average of almost 40% for the six in every seven terrestrial vertebrate species that have declined under human land uses [6], while well-monitored vertebrate populations have typically shrunk by almost 70% since 1970 [7]. All available indications are that these impacts will increase markedly this century [2,8].

Human activities harm biodiversity in numerous ways, including overharvesting, spreading non-native species, disrupting the climate and acidifying the oceans. Yet damaging species’ habitats—clearing them completely and degrading what is left to meet our demands for fibre, infrastructure, minerals, energy, recreation and especially food—is and for the foreseeable future will remain by a considerable measure the most important way in which we threaten other species [6,8–11]. To bend the curve, we therefore need to pay the greatest attention to the drivers of ongoing destruction and degradation of natural habitats, and then assess critically and open-mindedly what interventions offer most promise for mitigating them. This review is an attempt to challenge the thinking among many in conservation about how to address habitat loss. We look in particular at agriculture and its impacts on biodiversity. However, we suggest that many of our findings are likely to be relevant to other environmental outcomes (such as greenhouse gas emissions), and to other sectors where our use of resources harms other species; finding sustainable solutions for meeting humanity’s needs will require integrated analyses across many sectors and multiple outcomes of concern [12].

In the following section, we explain our focus on farming by examining its current and likely future impacts on wild species. We then present an overview of two widely advocated routes to reducing these impacts: shifting diets in richer countries towards less harmful foods and reducing food loss and waste. We consider which interventions appear promising, but conclude that while addressing diet and waste is essential for stemming the extinction crisis, supply-side approaches aimed at maintaining and increasing farm yields (that is, food production per unit area) are even more important. The notion that high-yield farming is critically important for delivering effective conservation may seem at first sight controversial, so we next examine the biological evidence for and against land sparing—pursuing sustainable high-yield farming in part of a landscape in order to safeguard or restore large areas of natural habitat elsewhere within it. In essence, the issue turns on the balance between the localized biodiversity benefits of lower yield production and the off-site biodiversity costs caused by the displacement of forgone production—a trade-off that we go on to argue needs to be considered in assessing the merits of almost any conservation intervention in potentially productive land. We use the penultimate section to examine two further practical concerns: how to deliver and sustain high-yield production at least cost to other outcomes of concern and how to ensure high-yield farming does indeed reduce pressure on natural habitats rather than stimulate further conversion. To close, we make five simple observations. While these may be uncomfortable, we nevertheless suggest they are key to making progress in slowing biodiversity loss.

## 2. The pre-eminence of farming

*‘If we fail on food, we fail on everything’ –* Charles Godfray, 2011

There are many reasons why people clear and degrade natural habitats—to extract timber, energy or minerals, to make way for infrastructure, through destructive fishing or hunting methods, or simply to assert tenure. However, making land available for crop and livestock production and further reducing its value for wild species through especially harmful ways of pursuing higher yields together constitute the greatest proximate drivers of habitat damage. Farming already takes up roughly 50% of all habitable land [13–15] and is continuing to expand. Forests are the main source of new farmland in the tropics [16], and at least 90% of recent tropical deforestation has been in landscapes where agriculture is the main driver, with roughly half of the newly cleared land then used for producing livestock or crops [17]. For cropland, independent global estimates confirm that expansion is not just continuing but accelerating [18,19].

**Figure 1.**
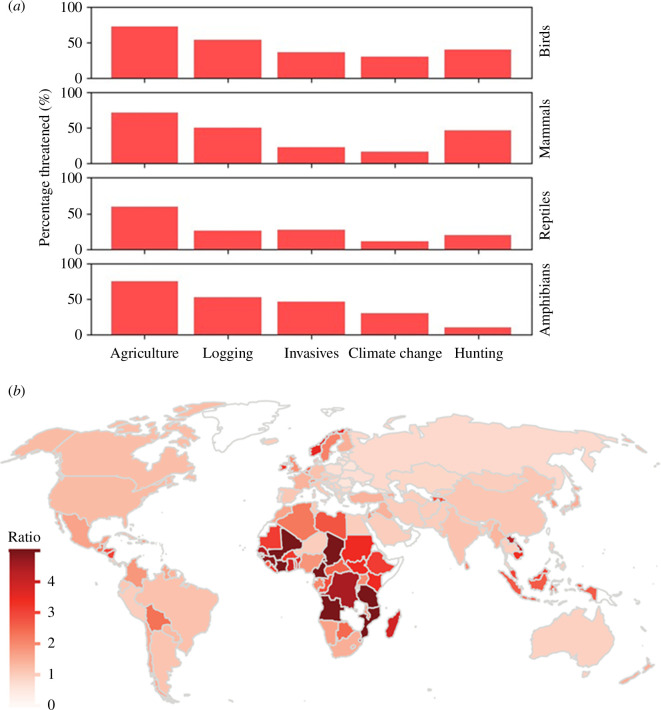
The pre-eminent importance of farming for conservation. (*a*) Agriculture threatens a greater percentage of threatened and near-threatened birds (1773 species), mammals (1280 species), reptiles (1468 species) and amphibians (2523 species) than any other human activity (data from IUCN [5]). (*b*) The area of cropland estimated to be needed by each country by 2060 compared to its cropland area in 2010, based on business-as-usual projections of population size, diet, trade and yield (from Tilman *et al.* [8]).

It is, therefore, no surprise that farming is the greatest threat to terrestrial biodiversity (figure 1*a*). According to the IUCN Red List [5], agriculture threatens 50–75% of all threatened and near-threatened amphibians, reptiles, mammals and birds—consistently more than any other human activity (although it is also worth noting that hunting—which impacts 46% of threatened and near-threatened mammals and is the greatest source of threat to assessed fish species [10]—is again driven largely by demand for food). Beyond biodiversity, the global food system uses >70% of all available freshwater [20], causes widespread eutrophication of rivers, lakes and coastal waters [21,22], and is by far the biggest reason for the worldwide application of >2M tonnes of pesticides each year, many of them potentially harmful to people as well as other species [23]. The global food system is also responsible for roughly one-third of all anthropogenic greenhouse gas emissions [24,25] with its critical importance for mitigating climate change even more apparent once the opportunity cost of forgone sequestration arising from continued land use is taken into consideration [26–28].

These impacts are set to worsen. Population growth and the increasing per capita impact of people’s diets as they become wealthier together mean that if current trends in farm yields continue, a large fraction of remaining natural habitats will be cleared for agriculture over the next three to four decades [8,29,30]. To meet rising food demand more cropland will be needed almost everywhere, but especially in sub-Saharan Africa (figure 1*b*; [8,31–33]). Here, according to one detailed analysis [8], a combination of chronic underinvestment in agriculture and resulting near-stagnant farm yields in many countries, rapid population growth and rising per capita demand together mean that unless current trajectories change, an area half the size of the continental United States may be cleared for new cropland between 2010 and 2060. Staggeringly, thirteen African countries are likely to need to expand their cropland area more than fourfold. The impacts of resulting habitat loss on biodiversity would be devastating, with a metric of extinction risk to large-bodied mammals, for example, projected to quadruple in 50 years [8].

Many other threats to biodiversity require mitigation. New demands on land to produce bioenergy and so-called ‘mass-timber’ for construction—which often draw on at best questionable claims of benefits to the global climate [34]—must be resisted. Other emergent problems—especially those arising from climate change—attract considerable attention in the literature and indeed the media [11]. Yet, this examination of current and indeed future threats indicates that the long-run fate of a great deal of biodiversity will be shaped, above all, by something much more prosaic: the pervasive and enduring challenge of how to meet humanity’s demands for food. So what options might we have for doing so with the least impact on other species?

## 3. Scope for shifting diets and cutting waste

*‘Had we but world enough, and time’ –* Andrew Marvell, early 1650s

Turning first to demand, one widely advocated approach for reducing the environmental costs of the food system is to shift diets—especially in richer countries and among more affluent people elsewhere—away from higher impact foodstuffs. The key motivation here is that some foods have much greater impacts than others [29,35,36]. Because they grow relatively slowly, produce methane and require a lot of land for feed or grazing, ruminant animals have especially high greenhouse gas footprints. Aggregate emissions per kilogram are thus on average around 40 times greater for ruminant meat than for pulses ([36,37]—although note the considerable within-commodity variation in emissions as well). The nitrogen-use efficiency, eutrophication and acidification potential of different foods tend (with important exceptions) to covary quite closely with their greenhouse gas footprint [35,38]. Effects on biodiversity are typically proxied by land use (for other proxies see [39,40]) with the area required per kilogram of product again typically much greater for ruminant-derived and some other animal proteins than for plant-based foodstuffs [36]. There is also extensive evidence that the meat-heavy diets typical of many western countries are linked to a higher prevalence of non-communicable diseases such as stroke, type 2 diabetes, coronary heart disease and colorectal cancer [29,41–43]. Thus, while there is of course an equally pressing need to improve access to nutritious food among billions living in poverty, elsewhere both human and planetary health would be much improved by a substantial shift towards more plant-based diets [8,29,32,37,40,41,43,44].

Many different interventions have been proposed for achieving this dietary transition. Evidence for the use of environmental labels is relatively weak; despite many experimental studies (reviewed by Potter *et al.* [45]), effect sizes in the few that have looked at real-world choices of different food types are modest [46]. In terms of prices, one field experiment that increased prices slightly for meat meals and reduced them for vegetarian meals also reported only a weak effect [47]. However, modelling work indicates that substantially increasing the relative price of meat through carbon taxation could lower its consumption considerably, and deliver sizeable health benefits [48]. Likewise, a large-scale experimental study in the UK suggests that a combined carbon and health tax on high greenhouse gas (GHG) footprint foods delivered in combination with information could cut food-related GHG emissions by over 15% [49]. That said, new taxes are politically unappealing, so changing relative prices by redirecting existing subsidies away from the livestock sector and into plant-based alternatives may be more feasible [50].

**Figure 2.**
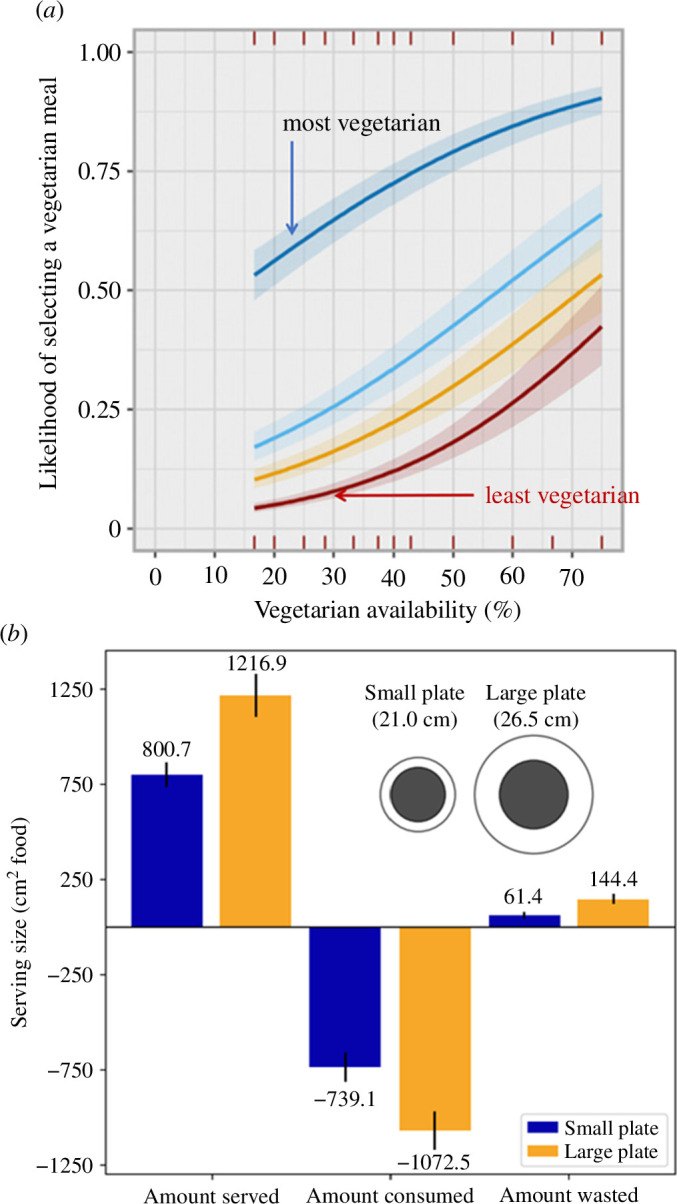
Promising interventions for reducing meat consumption and food waste. (*a*) Changes in the relative sales of vegetarian meals as their relative availability increases, with diners split by quartiles based on how often they chose vegetarian meals before the study [52]. Data based on 32 687 meal choices by 1394 individual diners at a Cambridge college. Note that total meal sales were unaffected by changes in meal availability and that ‘vegetarian’ here includes vegan meals. (*b*) In all-you-can-eat buffets diners using 26.5 cm diameter plates served themselves roughly 50% more food, ate 45% more and wasted more than twice as much as those using 21 cm plates [53]. The authors suggest customers choosing larger plates take and waste more food because they perceive a given serving to be smaller if it is on a bigger plate––a manifestation of the Delboeuf illusion, in which the black circle on the right appears smaller than that on the left. Food amounts were assessed visually, compared with plate area, and so are reported in cm^2^.

Interventions that alter consumer access to high-footprint meals also seem to offer promise. Entirely withdrawing ruminant meat from menus, for example, led to a 33% reduction in the per kilogram greenhouse gas footprint of the food sold by the University of Cambridge catering service [51]. Less drastic actions can work too. A large-scale observational and experimental study showed that doubling the relative availability of vegetarian or vegan meal choices in buffets (while always retaining a meat option) increased their relative sales by 40–80%, with the effect strongest among the most carnivorous diners (figure 2*a*; [52]; see also [54]). Plant-based meals have been successfully introduced as the default menu option on all lunches and dinners served at 11 New York City hospitals [55]; similar changes have resulted in strong effects in several experimental studies [56, 57]. However, moving items around in buffets, though a popular intervention, may be relatively ineffective: the only field experiment testing this idea found that switching meat options so they are placed last instead of first had limited impact, and none if buffets were short [58]. Although little studied, there is some evidence (reviewed in Boronowsky *et al.* [56]) that reformulating meals so they contain less meat reduces meat consumption. One last, potentially promising intervention might be to restrict advertising. A month-long ban on adverts for high-fat, salt and sugar products on the Transport for London network resulted in a striking 7% reduction in mean household calorie purchases across the capital [59]. Whether restricting meat advertising would be similarly effective is unknown, but a ban targeting meat promotion in the city of Haarlem is currently being implemented [60].

There is also considerable scope for reducing the environmental impact of the global food system by tackling food loss and waste. Estimates of their magnitude are hampered by poor data and inconsistent definitions, but food losses—those arising between harvest or slaughter up to (but not including) retail—are thought to account for around 14% of all potentially edible food [61]. Causes include pests and disease, aesthetic reasons, poor storage and transport infrastructure and wastage during processing. In the UK, cosmetic standards alone lead to the rejection of roughly 20% of all fresh fruit and vegetables [62]. On top of losses, food waste—incurred by retailers, food service providers and in households—means that an additional approximately 17% of all food produced for human consumption never reaches people’s mouths [63]. Contributory factors in this case include retailer marketing practices, excessive portions in restaurants, and consumer responses to best-before dates and sales offers. Given that loss and waste are typically greater for fresh fruit and vegetables [64], which people tend to buy more of as real incomes rise, some have argued that both may increase yet further [65].

As with shifting diets, many interventions have been devised to reduce food loss and waste. In food-service outlets, for example, behaviourally mediated actions include switching from disposable to ceramic plates and withdrawing trays so that customers have to carry their plates [66,67]. One remarkable study conducted in four all-you-can-eat buffets found that customers who chose plates that were 20% smaller in diameter on average served themselves 35% less food and wasted 57% less of what they took [53]—a result the authors attribute to the Delboeuf illusion, whereby a fixed-size circle appears smaller the larger a concentric circle around it becomes (figure 2*b*).

Approaches elsewhere in the food system include abandoning the cosmetic rejection of food; improving food storage, preservation and transport infrastructure; using better packaging to limit food spoilage; adopting more relaxed shelf-life standards; running education campaigns for householders; encouraging the use of kitchen diaries to record waste; and deploying food apps to facilitate sharing unwanted food before it expires [61,63–66]. Many of these are reported as being effective [63,65,66]—although interpreting the strength and external validity of findings is difficult because (as in the plate-size study) participants may self-allocate to particular treatments, there is commonly limited consideration of potentially confounding variables, and studies often rely on self-reporting to measure outcomes [65].

Nevertheless, there is some encouraging evidence that collectively these and other interventions can help. In the UK, the waste-reduction charity WRAP reported an overall 27% reduction in per capita post-farmgate losses and waste between 2007 and 2018 [68], suggesting the country was on track to meet its commitment under UN Sustainable Development Goal 12.3 of halving food waste by 2030. Progress elsewhere is less clear, however, and there is further concern that addressing loss and waste may trigger rebound effects. For instance, access to composting facilities may make supermarket managers or householders less concerned about reducing waste in other ways [63]. A recent economic framing of the problem suggests that consumption rebound from efficiency improvements and resulting price decreases could together more than halve the environmental benefits of cutting food loss and waste [69].

To summarize, shifting diets among wealthier consumers and cutting loss and waste across the food system can be achieved and are clearly vital if we are to slow the conversion of natural habitats. But there are clear indications that such measures will by themselves be nowhere near enough. Overall reductions in meat consumption, for example, are modest and potentially less than some estimates suggest [70,71]. And several global modelling exercises all conclude that significantly curbing farmland expansion over the next half-century cannot be achieved by bold changes in diets and/or food loss and waste alone [8,29,32,41,72]; see also [73]: to paraphrase Marvell, there is simply not enough space or time. Marked increases in farm yields are also essential—to meet greenhouse gas targets as well as slow land conversion—with biodiversity-focused models concluding that ambitious but feasible yield increases are even more important levers than shifting diets and lowering loss and waste [8,32].

## 4. The inescapable need for yield increases

‘*Whoever makes two ears of corn, or two blades of grass, to grow upon a spot where only one grew before, would deserve better of mankind, and do more essential service to his country, than the whole race of politicians put together’* – Jonathan Swift, 1726

The merits of high-yield production relative to other approaches to meeting people’s demand for food have been extensively explored in the land sharing/sparing debate. That literature is reviewed at length elsewhere [74–83], but to reprise the main arguments here, the idea behind land sharing is to increase biodiversity within farmland, while land sparing seeks to boost yields on already-cleared land in order to achieve the same overall level of production while making space for intact or restored natural habitats nearby. The chief challenge to land sharing is that most actions that make farmland more accommodating for biodiversity—limiting harmful fertilizers and pesticides, reducing farm specialization and retaining or reinstating microhabitat features such as field margins—typically tend to reduce farm yields. This is why farmers do not usually adopt them unless recompensed through subsidies or higher farmgate prices. It means that to reach any given level of production, sharing requires more area under farming, leaving less for natural habitats. The primary biological problem for sparing is the reverse—boosting yields almost always lowers biodiversity within farmed land. (Two other important challenges—maintaining high yields sustainably and limiting the spread of high-yield production—are discussed in §6.)

Which approach to organizing food production is likely to do least harm to biodiversity as a whole—sharing or sparing, or any other allocation of land across different yield regimes and to natural habitat—thus depends largely on two things: how biodiversity outcomes change as the agricultural yield of an area of land increases and the recognition that the amount of food produced in one place has inevitable consequences for the amount of production demanded elsewhere [12,75]. This in turn means that to be robust, empirical sharing/sparing assessments require three core elements [75,82,84].

1. Abundance-based measures of biodiversity of each of a large number of species. Studies that instead report just the presence or absence of species, or worse, simple species richness, lack essential information on the relative viability of populations and in the extreme risk failing to detect the replacement of specialist species by widespread generalists.
2. Data from climatically and edaphically matched sites across the full spectrum of yield regimes, including high-yielding systems, and critically, zero-yielding natural habitats present or potentially restorable in the study region.
3. Measures of yields in each site where biodiversity is assessed, so that relationships between abundance and yield can be quantified for each species, and in due course, the overall biodiversity consequences of contrasting scenarios for achieving any given level of production in a region can be compared without some scenarios creating additional but overlooked impacts via the displacement of forgone production elsewhere.

**Figure 3.**
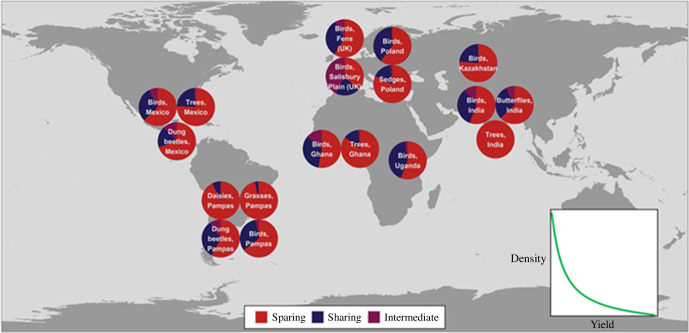
Summary of results from all sharing/sparing studies using the Green *et al*. [75] framework. Pie charts show proportions of species whose landscape-wide populations would be greatest under extreme land sparing (red), extreme sharing (blue) or any intermediate (purple); for most species their population density exhibits a negative convex relationship with increasing yield (inset), so their populations are largest under land sparing. Data are plotted for a total of 1766 individually assessed species; calculations assume present-day production levels. Image produced by Tom Finch.

To date, relatively few field studies have met all these criteria. However, those that do, report a striking pattern (figure 3). Of almost 2000 individually assessed species of birds, butterflies, beetles, trees, grasses, sedges and daisies whose population densities have been estimated across the full yield spectrum, the majority are sufficiently sensitive even to low-yield farming that they would fare better under land sparing than sharing or indeed any intermediate strategy [85–93]. In Europe, the presence of significant numbers of wild species associated with extensive farming means overall outcomes are slightly improved if some area is assigned to a third compartment, characterized by low-yield production tailored to the needs of those species ([90–92]; for a new and interesting suggestion that a low-yielding third compartment may also be useful in some regions for safeguarding agrobiodiversity, see [94]). But even here, the best-performing solutions all involve substantial increases in yields on most farmland in order to make space for this third compartment as well as for expanded natural habitats. In all these studies, sparing is even more important for threatened or narrowly distributed species, and becomes more beneficial in scenarios where (as is very likely) the demand for food production rises. Land sparing also outperforms sharing in studies using occupancy-based proxies for abundance [95], those measuring biodiversity in terms of phylogenetic or functional diversity [96–98] and indeed for several non-biodiversity outcomes, including above-ground carbon storage, nutrient cycling through dung removal, nature-based recreation and water quality [99–102].

Other empirical papers reach more mixed conclusions, variously reporting that biodiversity would fare better under land sharing, land sparing or more complex solutions[103–115]. However, each of these studies fails to meet one or more of the fundamental criteria we suggest are needed to make reliable inferences [75,82,84]: they use presence–-absence or species-richness measures of biodiversity, lack data from high-yielding systems and/or natural habitats, or draw conclusions across scenarios that are not matched in terms of overall production (often because there are no yield data), and hence ignore the consequences for biodiversity of having to meet shortfalls through increased production elsewhere. In contrast, to the best of our knowledge, every empirical study that does meet the criteria set out above reaches the same conclusion: that sparing would be biologically preferable to sharing, and hence that—alongside dietary change and waste reduction—achieving and sustaining high-yield farming is critical for slowing catastrophic habitat loss.

## 5. The risks of ignoring yields

*‘Every man takes the limits of his own vision for the limits of the world’*

*–* Arthur Schopenhauer, 1861

**Figure 4.**
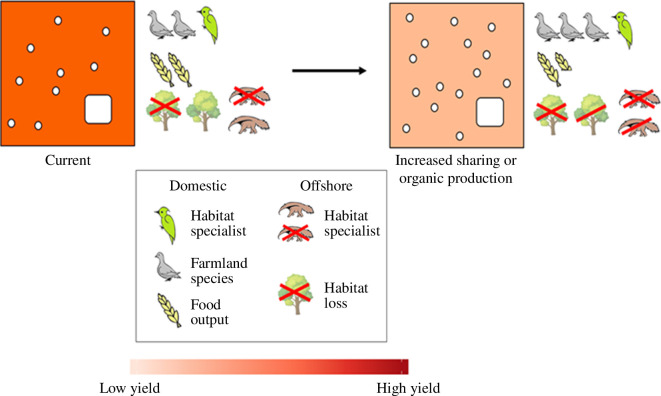
The likely effects of a typical European country increasing land sharing or organic production (re-drawn from Binner *et al.* [118]). This would probably increase the abundance of farmland species (woodpigeon icon) because of using fewer fertilizers and pesticides and reinstating some small habitat features (white circles), but would have little impact on domestic habitat specialists associated with large habitat blocks (woodpecker icon, white square). However, yields and hence food output (cereal icon) would fall. Forgone domestic production would then necessarily be met by increased production overseas, causing damage to habitats and specialist species overseas (anteater and tree icons). Note that the offshore impact of food imported into richer countries is typically far greater, on a per tonne basis, than that of domestic production (see text).

The importance of high-yield farming in making space for nature has significant implications for emerging land-use policies in Europe and beyond because of leakage—the displacement of production elsewhere because an intervention causes a shortfall in local supply (see also [12,116–118]). In response to NGO and public pressure, the UK government’s new Environmental Land Management scheme (ELMs) will substantially increase the allocation of subsidies to sharing-style agriculture [119]. In the European Union (EU), long-running environmental concerns have prompted the ‘farm to fork’ initiative, which aims by 2030 to cut fertilizer and pesticide use by 20% and 50%, respectively, and extend organic production to one-quarter of all farmland [120,121]. Japan is aiming for 25% of its farm area to be organic by 2050 [122]. At one level these commitments make sense. Introducing sharing-style and indeed organic farming is likely to increase the average abundance of farmland species (figure 4, woodpigeon icons; [123,124]). However, it will do little to help a country’s habitat specialists and will inevitably reduce productivity at the farm level (woodpecker and cereal icons)—best estimates for organic production, for example, are that this typically lowers yield by at least 19–25%, and indeed more once the areal requirements of fallow periods and green manure production are factored in [123–127]. Hence, reliance on imported food will rise.

The resulting increase in food production elsewhere will have profound consequences for the impacts these regions’ consumers have on global biodiversity (figure 4, tree and anteater icons). Increasing crop imports meant the EU already generated approximately 11 Mha of overseas deforestation from 1990 to 2014—most of it in Brazil and Indonesia [119]. The biodiversity cost of food imported into richer countries is very substantial [39,128] and often appears to exceed by some margin that of food produced domestically. Estimates derived using the commodityfootprints.earth dashboard, for example, indicate (based on methods in [129,130]) that the impact on extinctions of all food imported into the UK is roughly 2.1 times that of domestic production, on a per tonne basis; for wheat and potatoes the corresponding estimates are 1.1 and 2.0, respectively. Ignoring these sizeable but distant impacts—which signal that such policies will almost certainly cause net biodiversity harm—recalls Kahneman’s focusing illusion, that in focusing on one effect of a change we tend to overlook all its other effects [12,131].

Failure to consider the unintended but predictable effects of promoting lower yield farming methods can have human costs as well—as revealed in Sri Lanka in 2021, where a political desire to move the country to organic production prompted a near-instantaneous ban on importing chemical fertilizers and pesticides [132]. This quickly led to the halving of rice yields, yield collapses for other crops including key export commodities of 20–70%, and a food inflation rate of 95% per year. Together with a chronic fiscal deficit and the impacts of COVID-19 on international tourism, the agricultural crisis triggered anti-government protests, national bankruptcy and, in due course, the departure into exile of the president [132–134].

We suggest that similarly narrow thinking—the widespread failure of policymakers and indeed many within NGOs to consider the broader consequences of interventions that may appear locally benign—means that several major environmental initiatives beyond the farm sector may also be considerably less beneficial than intended (see also [12,118]). For example, China’s 1997 ban on domestic harvesting of natural forests in due course drove a 15% increase in imports [135], many of them from highly biodiverse tropical regions. The EU’s Biodiversity and Forest Strategies for 2030 seek to ban timber harvesting in all its remaining old-growth forests and impose yield-reducing practices in other domestic forests but are mute on how and where the resulting production shortfall will be met [136]. With imports from tropical forests already at a 15 year high there are grave concerns that these new restrictions will dramatically increase Europe’s consumption of much more biodiverse forests on other continents. And at a global scale, the agreement by signatories to the Convention on Biological Diversity to strive for 30% of all the Earth’s land surface to be conserved through protected areas and other effective area-based conservation measures by 2030 [137] may be laudable, but without equally bold commitments on farm and forest yields, it seems likely that the displacement of current production from newly conserved areas will lead to widespread habitat destruction in the remaining 70%. The literature indicates that such leakage of production as a result of efforts to protect or restore natural habitats is much more prevalent than is widely assumed [117]: it is the rule, not the exception. This in turn suggests that accounting for leakage when proposing interventions or assessing their effectiveness is essential and that reducing its prevalence by simultaneously promoting compensatory yield increases elsewhere will be critical to achieving overall conservation objectives [117,138].

## 6. Problems for achieving land sparing

*‘The best is the enemy of the good’ –* Voltaire, 1772

We now turn to two major practical challenges to realizing the potential conservation benefits of high-yield farming: delivering high yields sustainably in ways that do not increase environmental externalities and ensuring that more productive farming reduces pressure on land for nature rather than exacerbates it. Both topics have been reviewed extensively elsewhere [82,83,139–143], so here we summarize the major points rather than treating them exhaustively.

**Figure 5.**
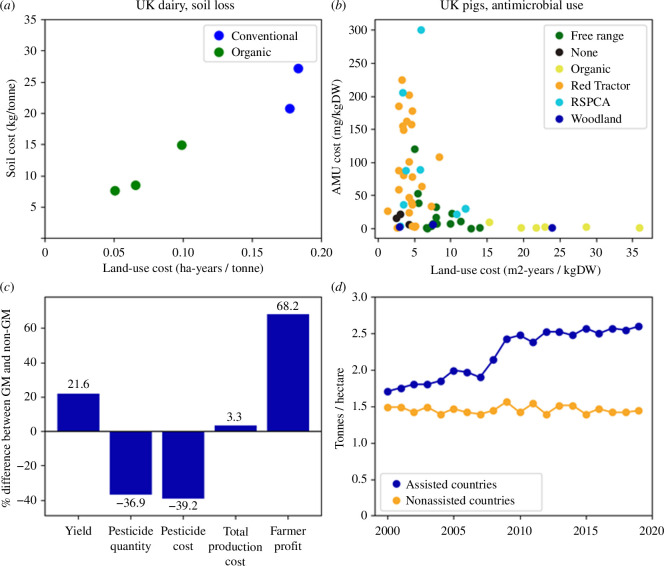
Delivering sustainable yield increases. (*a,b*) One framework for assessing the potential harms of higher yielding production methods is to plot externalities or other costs of a tonne of production under contrasting systems against how much land each requires [27]. Most such analyses to date reveal positive cost : cost associations—(*a*) as illustrated in a comparison of soil loss and land cost across conventional and organic UK dairy systems (from Balmford [27]). Negative associations, such as that between antimicrobial use and land cost across 74 UK pig production systems (*b*), instead indicate trade-offs [148]. These might potentially be addressed by identifying exceptional systems that are characterized by low costs in both domains and so lie in the bottom left of the plot; note these are not predicted by labelling schemes (colours). (*c,d*) Contrasting ways of achieving marked yield increases—the effects of (*c*) adopting GM soybean, maize and cotton, showing the percentage difference in outcomes compared with matched non-GM crops (from a meta-analysis by [149]); and (*d*) long-term international assistance to African farmers enabling them to access improved seeds and mineral fertilizers [150].

On the first issue, achieving and maintaining environmentally sustainable high-yield farming is tough: many practices are currently unsustainable in terms of their use of soil, water and other inputs and their release of nitrogen and phosphorus compounds, greenhouse gases and pesticides [144]. Moreover, yield growth is slowing, and for some key crops and regions, it is beginning to plateau [18,145]. However, in much of the world there are still sizeable gaps between average and already-attainable yields [146]: calculations for Brazil, for example, suggest narrowing current yield gaps there by just 25% would make sufficient space for the country to meet its exceptionally ambitious food and timber targets for 2040 without any expansion of the area under production [147]. Worldwide, many different approaches are being examined for their potential to close yield gaps and indeed to raise yield ceilings. In assessing their promise, it is also important to consider their likely impacts on environmental externalities and animal welfare (see figure 5*a,b* for two examples) and, of course, on smallholder farmers and rural livelihoods [151,152].

Much of the yield-enhancement literature is focused on the development and uptake of new varieties. Conventional breeding, augmented by technologies such as whole-genome screening and marker-assisted breeding, will continue to be critical for improving yields and livestock feed-conversion ratios, boosting photosynthetic and water-use efficiency and increasing tolerance to pests, diseases and environmental stresses [139,143,153,154]. Gene editing (GE) and genetic modification (GM) techniques can accelerate these improvements, alongside enhancing the nutritional value of crops, reducing requirements for pesticides and even improving animal welfare (figure 5*c*; [139,149,155,156]). Recent changes to regulatory regimes surrounding GE technologies have led to calls from the Royal Society and others for similar relaxation of restrictions on the farm-level deployment of GM crops and livestock [143].

There is also considerable potential to increase yields and reduce externalities through changes to farm practices—in many cases more swiftly than through genetic improvements [18]. At one extreme, vertical farming offers the promise of extraordinary increases in yields for high-value items such as salad vegetables [157,158]. But many other, much less capital-intensive approaches—including integrated pest management, push-pull methods of controlling pests, co-culture techniques, silvopasture and drip irrigation—are clearly capable of achieving marked increases in yields, often with lower inputs of water or potentially harmful chemicals [159–163]. Conservation agriculture and the broad array of methods now badged as regenerative farming also show promise in terms of restoring soils and enhancing water retention [164,165], although impacts on yields and lowering greenhouse emissions appear variable, and in some instances negative [166–169]. In contrast, for some other widely advocated approaches, such as organic farming, grass-fed livestock production and urban agriculture, most evidence to date suggests they are associated with substantial reductions in yields and increases (per tonne of production) in greenhouse gas emissions [35,123–127,170–173].

Perhaps the broadest conclusion is that there are often substantial opportunities to improve yields simply through adopting existing best practices. African smallholders, for example, commonly achieve dramatic increases in yields of staple crops when given access to improved seed varieties, modest amounts of inorganic fertilizer, and advice on how to improve soil fertility and structure [174–177]; the impact of sustained provision of such inputs is detectable even at continental scale (figure 5*d*; [150]). In China, the astonishingly ambitious rollout of locally tailored advice on cropping and the timing of inputs to 21 million smallholders led to an average increase in yields of maize, rice and wheat of >10% in 10 years, alongside an approximately 16% reduction in the release of reactive nitrogen [178]. And among oil palm producers in Ghana, adoption of a raft of simple improvements to harvesting practices, pruning and drainage regimes, and fertilizer and mulch application led over three years to a tripling of smallholder yields and an approximately 60% increase even in plantations [179]. The limiting factors to scaling up all of these achievements, it appears, are not technological or even regulatory; in very many cases farmers simply need sustained access to fertilizer, improved varieties, markets and sound agronomic advice.

However, the prospect of increasing yields raises the second major practical challenge in delivering land sparing: by lowering prices, raising profits or freeing up labour or capital, high-yield farming may stimulate increased production [180]. If such a rebound happens, sparing may still occur, but imperfectly—that is to say yield increases result in less than proportional reductions in the area under farming, and conservation gains are reduced. In the extreme case of a backfire or Jevons effect, greater yields increase production to such an extent that the area of farmed land grows; increasing yields in this case are then harmful to conservation. Large-scale empirical studies using cross-country panel regression or equilibrium models to investigate changes in areas under farming or natural habitats as yields increase present a mixed picture, in part because of differences in whether and how authors deal with confounding effects such as population growth (see reviews by [140,142]). A few studies [181,182] report that rebound effects are very sizeable, and others (e.g. [183]) that they are extremely small. However, most analyses report an intermediate degree of rebound, with effects stronger for cash crops than staples, and varying with how yield increases are achieved, with distance to markets and with governance regimes; Jevons effects are rare [184–192].

Smaller scale observations of how land cover changes with yield confirm this broad conclusion. In the Chaco of northern Argentina, for example, market-driven investments in soy were associated with very marked increases in agricultural yields but reductions in deforestation [193]. In the Peruvian Amazon, villages with greater access to fertile floodplain soils earn more cash from rice production and clear less old-growth forest than other villages [194]. Results from two yield-enhancement programmes in Africa underscore the context dependence of land sparing. Farmers receiving support to intensify maize production in Malawi slowed their rate of forest clearance, but there was no such slowdown among farmers receiving coupons for intensifying tobacco production [175]. And in Zambia, subsidized access to improved maize seeds generally slowed forest conversion, but access to fertilizers had a weakly opposite effect [177].

**Figure 6.**
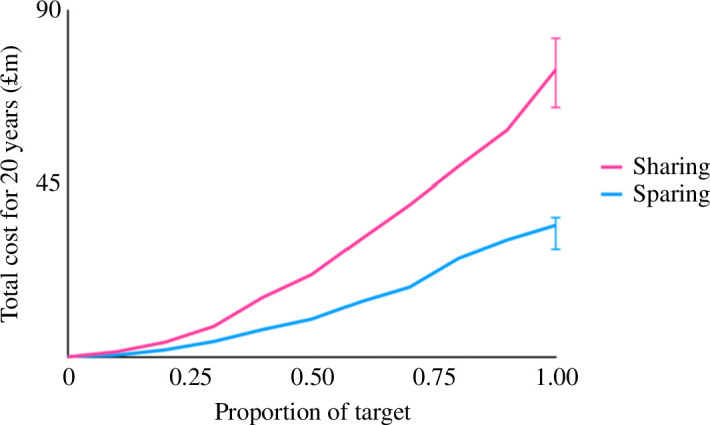
Potential cost savings from land sparing. Lines show estimated overall costs to the UK taxpayer (combining compensation payments and capital, administration and monitoring costs) of 20 year sharing and sparing schemes scaled to deliver a combined biodiversity and carbon target. Ninety-five per cent bootstrapped confidence intervals reflect uncertainty in compensation payments [201].

In general, it thus appears that yield increases do typically spare land, albeit imperfectly. But importantly, these observations describe what has been termed ‘passive’ sparing, mediated essentially by market effects alone [195]. Re-analyses of data from Ghana and India indicate sparing still outperforms land sharing unless levels of rebound exceed approximately 70% [196]. However, active sparing interventions, which seek to couple yield increases directly with habitat retention or restoration have the potential to reduce rebound effects substantially, and so enhance land sparing. Possible coupling mechanisms include:

1. *Strict land-use zoning.* Regulating where farming occurs in order to incentivize intensification and hence yield increases there while safeguarding more intact areas. As one example, formalizing land rights in Benin simultaneously encouraged yield-enhancing investments by smallholders and cut deforestation by one-fifth [197].
2. *Spatially strategic deployment of farmer support.* Targeting low-yielding but already-cleared areas and avoiding conversion frontiers. Examples include installing irrigation for lowland but not upland rice farmers in Palawan—upland farmers instead chose to work on lowland fields, slowing upland deforestation [198]; and delivery of technical support to eastern Amazonian smallholders, which doubled their incomes while reducing forest clearance by 80% [199].
3. *Creating market instruments.* Making access to high-value markets or credit conditional on conservation outcomes. Around Sierra Leone’s Gola forest, for instance, cocoa farmers given access to overseas markets (as well as technical training) markedly reduced their deforestation activities [200].
4. *Providing direct financial support.* Offering grants or subsidies that are conditional on simultaneous delivery of high yields and sizeable areas of natural habitat. As one illustration of the potential benefits of this approach, a choice experiment with subsidized UK farmers found that although they would require higher payments for sparing compared with sharing interventions, the conservation benefits these deliver are so much greater that overall costs to the public would be halved (figure 6; [201]).

Obviously, each of these mechanisms is only relevant in particular socio-economic and political circumstances. Other devices for linking yield increases to the creation of natural habitats will be needed, and it is unlikely that any will deliver perfect sparing. Likewise, high-yield farming will impose some environmental and perhaps other costs (such as reduced livestock welfare), although for those outcomes measured to date, perhaps fewer—expressed per tonne of production—than are sometimes assumed (figure 5*a,b*; [27,148,202]). We need to continue to develop and identify high-yield systems that reduce those negative outcomes as far as possible. Yet although this will be challenging, we contend that working with agricultural producers, researchers and regulators to deliver sparing through sustainable high-yield production will deliver far greater conservation gains than focusing on lower yielding strategies that will condemn many remaining natural habitats to conversion.

## 7. Concluding remarks

Five overarching observations emerge from this review:

1. While other drivers attract more attention, the mundane challenge of meeting human food demands remains the pre-eminent threat to wild nature.
2. Reducing demand for higher footprint foods and cutting food loss and waste are essential, but by themselves insufficient to avert catastrophic habitat loss: we also need to change how we grow our food.
3. Most of >2000 species investigated in detail depend on natural habitats and decline sharply under any sort of farming, so rather than attempting to accommodate wildlife within farms at the cost of needing more land for food we need instead to focus on increasing farm yields sustainably in order to make space beyond farms for sizeable areas of habitat.
4. Because intervening to conserve nature in farming landscapes almost always reduces yields, we must always consider the impacts of meeting the resulting forgone production elsewhere before concluding whether our actions are likely to be beneficial or cause more overall harm than good.
5. To limit negative externalities and rebound effects, work needs to be done to identify, promote and regulate sustainable high-yield farming—which in turn requires the open-minded and ambitious commitment of government, NGOs, researchers and the private sector.

Several of these observations will, we suspect, be uncomfortable for conservationists. They challenge conservation orthodoxy, raise significant technical, regulatory and indeed political problems [203], and require us to work much more closely with actors we might consider part of the problem, not key contributors to the solution. Agriculture itself is not the problem: at a fundamental level, it is that we will shortly number 10 billion increasingly demanding people, while in a more proximate sense, the problem is that extensive wildlife-friendly farming is making things much worse. We of course urgently need to shift away from high-footprint diets and cut food waste, but we maintain that unless we also undertake difficult and perhaps counterintuitive supply-side actions as well, centred on the land-efficient production of food, we will fail in our efforts to bend the curve of biodiversity loss.

‘*All the evidence to date is that when societies put their mind to solving a problem, they can generally do it … I think the challenge is to break the problems down into manageable chunks and solve them—being careful not to set aside the difficult and important ones*.’

– Georgina Mace, 2009

## Data Availability

This article has no additional data.
